# Utility of PREDICT-HF score in high-risk Asian heart failure patients receiving sacubitril/valsartan

**DOI:** 10.3389/fcvm.2022.950389

**Published:** 2022-07-25

**Authors:** Chien-Yi Hsu, Hung-Yu Chang, Chieh-Ju Chao, Wei-Ru Chiou, Po-Lin Lin, Fa-Po Chung, Wen-Yu Lin, Jin-Long Huang, Huai-Wen Liang, Chia-Te Liao, Ying-Hsiang Lee

**Affiliations:** ^1^Division of Cardiology and Cardiovascular Research Center, Department of Internal Medicine, Taipei Medical University Hospital, Taipei, Taiwan; ^2^Division of Cardiology, Department of Internal Medicine, School of Medicine, College of Medicine, Taipei Medical University, Taipei, Taiwan; ^3^Taipei Heart Institute, Taipei Medical University, Taipei, Taiwan; ^4^Heart Center, Cheng Hsin General Hospital, Taipei, Taiwan; ^5^Faculty of Medicine, School of Medicine, National Yang Ming Chiao Tung University, Taipei, Taiwan; ^6^Department of Cardiovascular Diseases, Mayo Clinic Rochester, Rochester, MN, United States; ^7^Department of Medicine, MacKay Medical College, New Taipei, Taiwan; ^8^MacKay Junior College of Medicine, Nursing and Management, Taipei, Taiwan; ^9^Division of Cardiology, Taitung MacKay Memorial Hospital, Taitung, Taiwan; ^10^Division of Cardiology, Department of Internal Medicine, Hsinchu MacKay Memorial Hospital, Hsinchu, Taiwan; ^11^Division of Cardiology, Department of Medicine, Taipei Veterans General Hospital, Taipei, Taiwan; ^12^Division of Cardiology, Department of Medicine, Tri-Service General Hospital, National Defense Medical Center, Taipei, Taiwan; ^13^Department of Medical Education, Taichung Veterans General Hospital, Taichung, Taiwan; ^14^Department of Post-Baccalaureate Medicine, College of Medicine, National Chung-Hsing University, Taichung, Taiwan; ^15^Division of Cardiology, Department of Internal Medicine, E-Da hospital, I-Shou University, Kaohsiung, Taiwan; ^16^Division of Cardiology, Chi-Mei Medical Center, Tainan, Taiwan; ^17^Cardiovascular Center, MacKay Memorial Hospital, Taipei, Taiwan; ^18^Department of Artificial Intelligence and Medical Application, MacKay Junior College of Medicine, Nursing, and Management, Taipei, Taiwan

**Keywords:** heart failure, sacubitril/valsartan, PREDICT-HF model, high-risk population, TAROT-HF

## Abstract

**Objective:**

The aim of this study was to investigate the application of sacubitril/valsartan in clinical practice and the utility of PREDICT-HF score for outcome prediction in Asian heart failure patients with difference risk profiles.

**Methods:**

The TAROT-HF study was a multicenter, single-arm, observational study. Totally 1,187 outpatients with HFrEF treated with sacubitril/valsartan were enrolled and categorized by: (1) high-risk group with ≥1 of the following three risk factors: old age (≥80 years), low baseline systolic blood pressure (<100 mmHg), and renal impairment (eGFR <30 ml/min/1.73 m^2^), and (2) standard-risk group, those who did not have any risk factors. Clinical outcomes were assessed using the PREDICT-HF risk model.

**Results:**

A total of 305 (25.7%) patients matched the criteria for the high-risk group. The event rates of cardiovascular death or first unplanned heart failure hospitalization (HFH) among the overall population, high-risk, and standard-risk groups were 13.7, 24.9, and 10.8 events per 100 patient-years, respectively. The C statistics for the PREDICT-HF model in the overall cohort and high-risk group for cardiovascular death or first unplanned HFH at 2 years were 0.73 (95% CI 0.70–0.76) and 0.71 (95% CI 0.65–0.76), respectively. The permanent discontinuation rate among the high-risk patients was significantly higher than that among the standard-risk patients (8.3 vs. 2.5 per 100 patient-years, *p* < 0.001).

**Conclusions:**

Real-world outcomes of the TAROT-HF study demonstrated that the PREDICT-HF model performed well in Asian HFrEF patients. Three easily detected clinical profiles of age, renal function, and systolic BP could help to identify patients at risk before initiating sacubitril/valsartan.

## Introduction

Heart failure (HF) is associated with high morbidity, mortality, and prolonged and frequent hospitalizations, leading to a significant burden on health care systems worldwide (1). Evidence-based medical therapy, including renin-angiotensin system inhibitors (RASis), mineralocorticoid receptor antagonists (MRAs) and cardio-selective beta-blockers, is the most effective way to reduce mortality and morbidity of patients with heart failure and reduced ejection fraction (HFrEF) (2–8). The Prospective Comparison of ARNI with ACEI to Determine Impact on Global Mortality and Morbidity in Heart Failure (PARADIGM-HF) trial established the beneficial effect of using sacubitril/valsartan (Sac/Val) over RASis for HFrEF patients (9). Among Asian population, the Prospective comparison of ARNI with ACEi to determine the noveL beneficiaL trEatment vaLue in Japanese Heart Failure patients (PARALLEL-HF) study applied similar inclusion and exclusion criteria as the PARADIGM-HF study, and demonstrated that Sac/Val was well-tolerated in Japanese HFrEF patients (10, 11).

However, several limitations have been raised regarding the generalizability of the PARADIGM-HF trial and study population with regards to its representativeness of real-world HFrEF patients (12). For example, patients only qualified for randomization if they were normotensive, had an estimated glomerular filtration rate (eGFR) ≥30 ml/min/1.73 m^2^, and were able to tolerate a run-in period of enalapril 20 mg daily and a target dose of Sac/Val. Real-world patients are usually older, more fragile, and have extensive comorbidities (13). In addition, in real-world practice, hypotensive and renally impaired patients generally have higher risks than patients with normal blood pressure and renal function. However, these patient groups were under-represented in the PARADIGM-HF and PARALLEL-HF studies. Currently, long-term safety and effectiveness data from unselected Asian patients are limited.

Recently, a new prognostic prediction tool for patients with HFrEF, the PREDICT-HF (Risk of Events and Death in the Contemporary Treatment of Heart Failure) model (14), was developed using the PARADIGM-HF trial cohort and validated in other large data sets to predict mortality and morbidity. Sac/Val has been reimbursed by the Taiwanese National Health Insurance system for the treatment of HFrEF since March 2017. Therefore, the aim of this study was to investigate the application of Sac/Val in clinical practice and the utility of PREDICT-HF model for outcome prediction in Asian heart failure patients with difference risk profiles.

## Methods

### Study design

The TAROT-HF (*T*reatment with *A*ngiotensin *R*eceptor neprilysin inhibitor f*O*r *T*aiwan *H*eart *F*ailure patients) study was a principal investigator-initiated, multicenter, real-world observational study of HFrEF patients in Taiwan. Outpatients with HFrEF who began treatment with Sac/Val were enrolled from March 2017 to December 2018. According to the Food and Drug Administration label in the given country, Sac/Val is indicated for symptomatic chronic heart failure (New York Heart Association Class II-IV symptoms) and reduced ejection fraction [left ventricular ejection fraction (LVEF) ≤ 40%]. The study design, purpose, and rationale have been described in detail in the protocol paper and previous studies (15–17). Each hospital's institutional ethics committee approved this study, which complied with the ethical principles of the Declaration of Helsinki.

In brief, a total of 1,772 HFrEF patients who initiated Sac/Val treatment at 10 hospitals between March 2017 and December 2018 were consecutively enrolled and analyzed. The eligibility criteria were: (1) age >20 years, (2) a diagnosis of symptomatic HF with NYHA Fc II-IV symptoms, (3) with documented echocardiographic LVEF ≤ 40%, and (4) first treatment with any dose of Sac/Val. Among the 1,772 patients, the clinical outcomes of 585 patients who initiated Sac/Val treatment during an acute decompensated HF hospitalization (TAROT-AHF arm) have been reported (18). In the present study, we analyzed the other 1,187 patients who initiated Sac/Val at an outpatient department (TAROT-CHF arm).

The patients were further classified into high-risk and standard-risk groups. High-risk patients were defined as those who had one or more of the following three risk factors: old age (≥80 years), low systolic blood pressure (<100 mmHg), and renal impairment (eGFR <30 ml/min/1.73 m^2^). Outcomes and clinical data, including cardiovascular death, all-cause mortality, HF hospitalization (HFH), and permanent discontinuation of Sac/Val were collected from the date of initiating Sac/Val to March 31, 2021.

### Utilization of the PREDICT-HF model

The details of the PREDICT-HF risk model have been published previously (14). In brief, Simpson et al. performed multivariate analysis of the PARADIGM-HF study cohort, and identified several variables that could predict cardiovascular death, all-cause mortality, and the composite of cardiovascular death or HFH at both 1 and 2 years. Data from the TAROT-HF study were entered into the online calculator (http://www.predict-hf.com) to calculate the risk for each patient. Estimated outcomes at 1 and 2 years were examined and compared with observed clinical outcomes.

### Statistical analysis

Continuous variables were expressed as mean value ± standard deviation, and categorical variables were reported as percentages. Differences in baseline characteristics and clinical parameters in the high-risk and standard-risk groups were tested using the chi-square test for categorical variables. The student's *t*-test or the Wilcoxon rank-sum test was used for comparisons of continuous data. The risks of cardiovascular death, all-cause mortality, and composite of cardiovascular death or HFH were analyzed using survival analysis with the Kaplan–Meier method and log–rank test. Multivariate Cox regression analysis was performed to assess the factors associated with clinical outcomes. The predicted vs. actual outcomes at 1 and 2 years were compared in quartiles of the PREDICT-HF risk scores. The C statistic was used to assess the discriminative ability of the PREDICT-HF model when applied to the TAROT-HF cohort to estimate outcomes at 1 and 2 years. A *p*-value of <0.05 was considered to be statistically significant. The statistical analyses were performed using IBM SPSS Statistics software version 24.0 (IBM SPSS, IBM Corp, Armonk, NY, USA).

## Results

### Baseline characteristics and treatments

A total of 1,187 HFrEF patients (mean age 61.7 ± 14.3 years, 76.3% male, mean LVEF 29.4 ± 7.1%) who initiated Sac/Val at an outpatient department from 10 hospitals between 2017 and 2018 were included in this study. Among these patients, 305 (25.7%) fulfilled the criteria for the high-risk group (253 had only one risk factor; 52 had two or three risk factors), and 882 (74.3%) patients did not match the high-risk criteria (standard-risk group). Table 1 shows detailed baseline characteristics of the study cohort. The high-risk patients were significantly older, more predominantly female, had lower body mass index, eGFR and systolic blood pressure and more severe HF symptoms, and they tended to have a history of hypertension, atrial fibrillation, HF hospitalization, peripheral arterial disease, chronic obstructive pulmonary disease, kidney disease, thyroid disease, malignancy, and hyperuricemia than the standard-risk patients.

**Table 1:** Demographics, clinical characteristics and treatment of the current study and clinical trials of sacubitril/valsartan.

	**TAROT-CHF overall** ***N* = 1,187**	TAROT-CHF high-risk^*^ ***N* = 305**	**TAROT-CHF standard-risk** ***N* = 882**	**PARADIGM-HF** ***N* = 8,442**	**PARALLEL-** **HF** ***N* = 225**
Age, years	61.7 ± 14.3	70.1 ± 16.0	58.9 ± 12.5	63.7	67.9
≥80, %	10.4	40.3	0.0	7.0	10.7
≥75, %	20.0	47.2	10.5	18.5	26.9
Female sex, %	23.7	32.5	20.6	21.9	14.2
Body mass index, kg/m^2^	25.7 ± 4.9	24.3 ± 4.6	26.2 ± 5.0	28.1	24.5
eGFR, ml/min/1.73 m^2^	66.0 ± 29.4	43.6 ± 27.6	73.7 ± 25.8	68.1	57.9
<60 ml/min/1.73 m^2^, %	40.1	69.5	29.9	35.3	28.0
<30 ml/min/1.73 m^2^, %	10.8	42.0	0.0	0.0	0.0
Systolic blood pressure, mmHg	123.4 ± 19.1	114.1 ± 22.2	126.6 ± 16.7	128.4	122.3
<100 mmHg, %	9.3	36.1	0.0	0.0	0.0
LVEF, %	29.4 ± 7.1	30.1 ± 7.6	29.2 ± 6.9	29.5	28.1
Ischemic etiology, %	42.5	45.9	41.3	59.9	47.6
NYHA Fc III/IV, %	26.1	34.8	23.1	35.0	6.2
Co-morbidities					
Hypertension, %	51.9	58.0	49.8	70.7	67.6
Diabetes mellitus, %	41.1	44.9	39.8	34.5	46.2
Prior myocardial infarction, %	29.1	28.9	29.3	43.2	43.1
Prior stroke, %	11.2	12.5	10.8	8.6	9.3
Atrial fibrillation, %	32.2	42.6	28.6	36.5	33.8
Prior HF hospitalization, %	58.8	68.2	55.6	62.8	72.9
PAD, %	6.1	11.8	4.1	5.8	NR
COPD, %	9.2	13.4	7.7	12.8	NR
History of renal disease, %	29.1	54.4	20.3	17.2	NR
Prior thyroid disease, %	7.2	10.2	6.2	NR	NR
Hyperuricemia, %	17.4	18.0	17.1	NR	NR
History of malignancy, %	6.5	10.2	5.2	4.9	NR
Treatment, %					
ACEi/ARB/ARNI	100.0	100.0	100.0	100.0	100.0
Beta-blocker	80.7	72.8	83.4	94.3	94.7
MRA	62.0	51.1	65.8	58.4	59.1
Digoxin	20.4	20.7	20.3	30.8	8.4
CRT	6.1	7.5	5.7	6.8	12.4
ICD	7.8	6.6	8.3	14.8	6.7

* *Definition of high-risk patients: those who had one or more of the following three risk factors: old age (≥80 years), low baseline systolic blood pressure (<100 mmHg), and renal impairment (estimated glomerular filtration rate <30 ml/min/1.73 m^2^)*.

*ACEi, angiotensin-converting-enzyme inhibitors; ARB, angiotensin II receptor blockers; ARNI, angiotensin receptor-neprilysin inhibitor; COPD, chronic obstructive pulmonary disease; CRT, cardiac resynchronization therapy; eGFR, estimated glomerular filtration rate; HF, heart failure; ICD, implantable cardioverter defibrillator; LVEF, left ventricular ejection fraction; MRA, mineralocorticoid receptor antagonists; NYHA, New York Heart Association; PAD, peripheral arterial disease*.

Table 1 also shows the baseline characteristics of two randomized trials of Sac/Val (PARADIGM-HF and PARALLEL-HF trials). Among the high-risk TAROT-CHF patients, 42.0% had an eGFR <30 ml/min/1.73 m^2^ and 36.1% had systolic blood pressure <100 mmHg. These patients were generally excluded from the PARADIGM-HF and PARALLEL-HF trials. The proportions of elderly and female patients were significantly higher in the high-risk TAROT-CHF cohort than the PARADIGM-HF and PARALLEL-HF trials.

### Clinical outcomes

**Figure 1:**
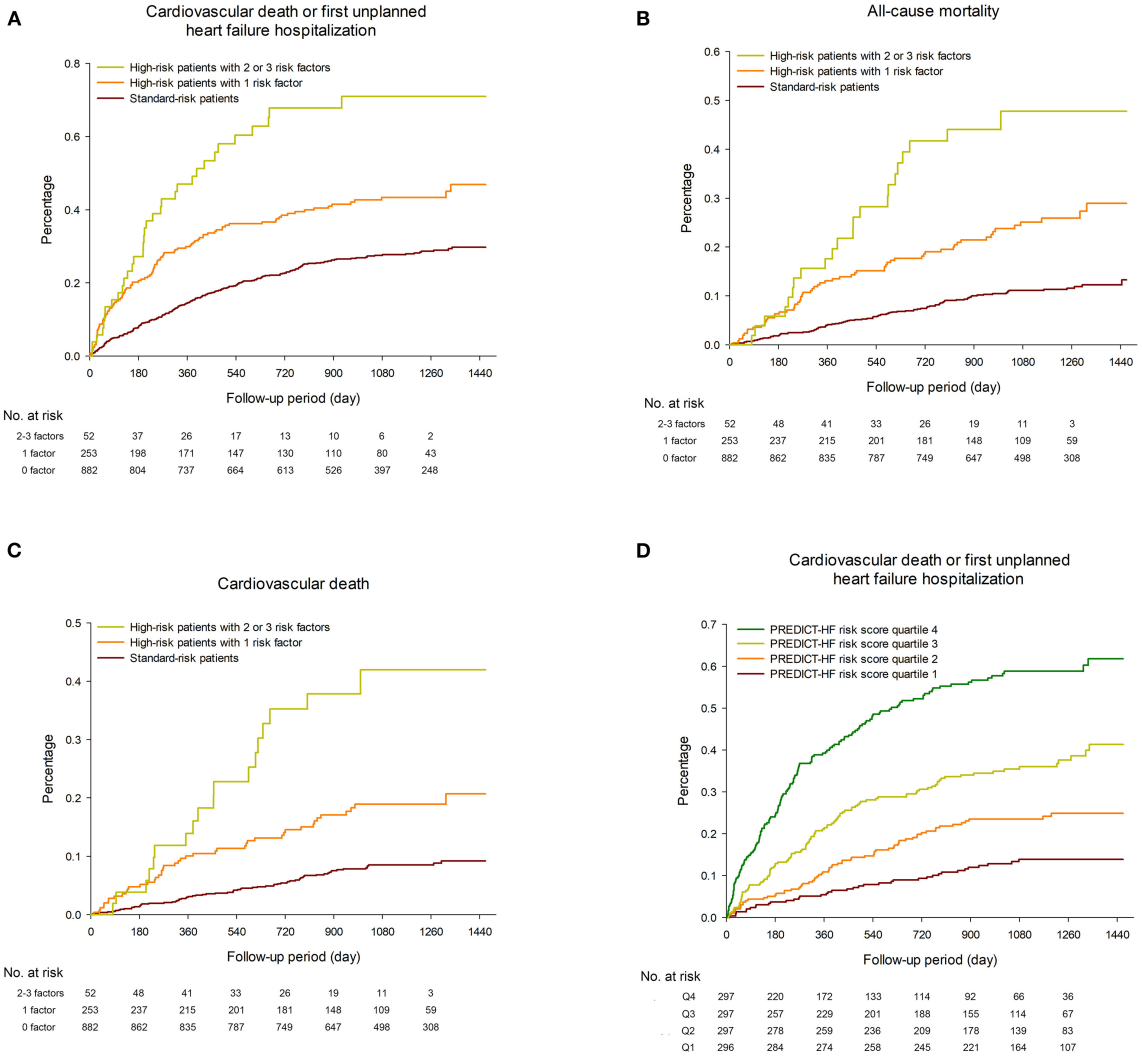
Kaplan–Meier survival curves for **(A)** cardiovascular death or first unplanned heart failure hospitalization (HFH), **(B)** all-cause mortality and **(C)** cardiovascular death among the study population, stratified by different risk groups. **(D)** Kaplan–Meier plots of the observed event rate in the TAROT-CHF cohort for clinical outcomes, categorized by quartile of PREDICT-HF risk score.

Figure 1 shows Kaplan–Meier survival curves for clinical outcomes among the study population. Table 2 shows the event rates of the current study and other recently published randomized trials. During a median follow-up period of 36.7 months (IQR 27.5–43.3 months), the event rate of cardiovascular death or first unplanned HFH among the overall population was 13.7 events per 100 patient-years, including 10.8 and 24.9 per 100 patient-years in the standard-risk and high-risk groups, respectively. Among the high-risk patients, the event rates of those with only one risk factor and those with two or more risk factors were 21.4 and 51.6 per 100 patient-years, respectively (Figure 1A, *p* < 0.001). The incidence rates of all-cause mortality and cardiovascular death among the overall TAROT-CHF cohort were 5.5 and 4.0 per 100-person years, respectively (Figures 1B,C).

**Table 2:** Comparisons of outcomes among different randomized controlled trials and the current study.

	**Active arm**		**Placebo/comparator**	
	***n* (%)**	**Events/100 patient-years**	***n* (%)**	**Events/100 patient-years**
**Cardiovascular death or heart failure hospitalization**				
PARADIGM-HF	914 (21.8)	10.5	1,117 (26.5)	13.2
DAPA-HF	382 (16.1)	11.4	495 (20.9)	15.3
EMPEROR-reduced	361 (19.4)	15.8	462 (24.7)	21.0
VICTORIA	897 (35.5)	33.6	972 (38.5)	37.8
TAROT-CHF, overall	378 (31.8)	13.7		
TAROT-CHF, high-risk	140 (45.9)	24.9		
TAROT-CHF, standard-risk	238 (27.0)	10.8		
**Cardiovascular death**				
PARADIGM-HF	558 (13.3)	6.0	693 (16.5)	7.5
DAPA-HF	227 (9.6)	6.5	273 (11.5)	7.9
EMPEROR-reduced	187 (10.0)	7.6	202 (10.8)	8.1
VICTORIA	414 (16.4)	12.9	441 (17.5)	13.9
TAROT-CHF, overall	132 (11.1)	4.0		
TAROT-CHF, high-risk	62 (20.3)	8.4		
TAROT-CHF, standard-risk	70 (7.9)	2.7		
**All-cause mortality**				
PARADIGM-HF	711 (17.0)	7.6	835 (19.8)	9.0
DAPA-HF	276 (11.6)	7.9	329 (13.9)	9.5
EMPEROR-reduced	249 (13.4)	10.1	266 (14.2)	10.7
VICTORIA	512 (20.3)	16.0	534 (21.2)	16.9
TAROT-CHF, overall	180 (15.2)	5.5		
TAROT-CHF, high-risk	83 (27.2)	11.3		
TAROT-CHF, standard-risk	97 (11.0)	3.8		

*Median follow-up period: PARADIGM-HF 27 months; DAPA-HF 18.2 months; EMPEROR-Reduced 16 months; VICTORIA 10.8 months; TAROT-CHF 36.7 months*.

Table 3 lists the parameters associated with clinical outcomes. The patients with two or more risk factors had significantly worse outcomes than those with only one risk factor, and the patients without any risk factors had a better prognosis. These differences remained significant after multivariate Cox regression analysis.

**Table 3:** Multivariate analysis for high-risk vs. standard-risk patients and clinical outcomes.

	**Univariate analysis**			**Multivariate analysis**		
	**Hazard ratio**	**95% confidence interval**	***p*-value**	**Hazard ratio**	**95% confidence interval**	***p*-value**
Cardiovascular death or first unplanned hospitalization for heart failure						
Standard-risk	1	–	–	1	–	–
High-risk (only 1 risk factor)	1.87	1.49–2.36	<0.001	1.62	1.28–2.05	<0.001
High-risk (2 or 3 risk factors)	3.81	2.66–5.47	<0.001	2.98	2.07–4.31	<0.001
NYHA Fc III/IV	2.30	1.87–2.82	<0.001	2.19	1.78–2.70	<0.001
Diabetes mellitus	1.44	1.18–1.76	<0.001	1.34	1.09–1.65	0.005
Prior stroke	1.56	1.18–2.07	0.002	1.35	1.01–1.80	0.044
COPD	1.85	1.37–2.49	<0.001	1.60	1.18–2.17	0.002
Prior thyroid disease	1.74	1.26–2.41	0.001	1.69	1.22–2.36	<0.001
Hyperuricemia	1.55	1.22–1.96	<0.001	1.53	1.20–1.95	0.001
Prior HF hospitalization	2.35	1.86–2.95	<0.001	2.00	1.58–2.53	<0.001
ICD implantation	1.99	1.47–2.69	<0.001	1.80	1.33–2.45	<0.001
Cardiovascular death						
Standard-risk	1	–	–	1	–	–
High-risk (only 1 risk factor)	2.47	1.69–3.60	<0.001	2.21	1.50–3.26	<0.001
High-risk (2 or 3 risk factors)	5.98	3.55–10.05	<0.001	4.74	2.73–8.23	<0.001
NYHA Fc III/IV	2.45	1.74–3.45	<0.001	1.76	1.22–2.53	0.002
Prior thyroid disease	2.06	1.25–3.38	0.005	1.70	1.01–2.86	0.048
Peripheral arterial disease	3.04	1.89–4.90	<0.001	2.10	1.29–3.44	0.003
ICD implantation	2.21	1.39–3.52	0.001	1.93	1.20–3.11	0.007
LVEF	0.95	0.92–0.97	<0.001	0.96	0.93–0.98	<0.001

*Multivariate analysis was adjusted for sex, heart failure etiology, body mass index, left ventricular ejection fraction, New York Heart Association functional class, history of heart failure hospitalization, atrial fibrillation, hypertension, diabetes, chronic obstructive pulmonary disease, peripheral arterial disease, prior stroke, history of thyroid disease, hyperuricemia, history of malignancy, device therapies, and prescriptions of heart failure medications*.

*COPD, chronic obstructive pulmonary disease; CRT, cardiac resynchronization therapy; HF, heart failure; ICD, implantable cardioverter defibrillator; NYHA, New York Heart Association*.

During follow-up, a total of 637 HFH events occurred in 330 patients. The incidence of total HFH events was 19.3 per 100-person years, including 75.3, 29.4, and 14.6 per 100-person years for those with two or three risk factors, one risk factor, and the standard-risk patients, respectively (*p* < 0.001).

### Predicting risks using the PREDICT-HF model

**Figure 2:**
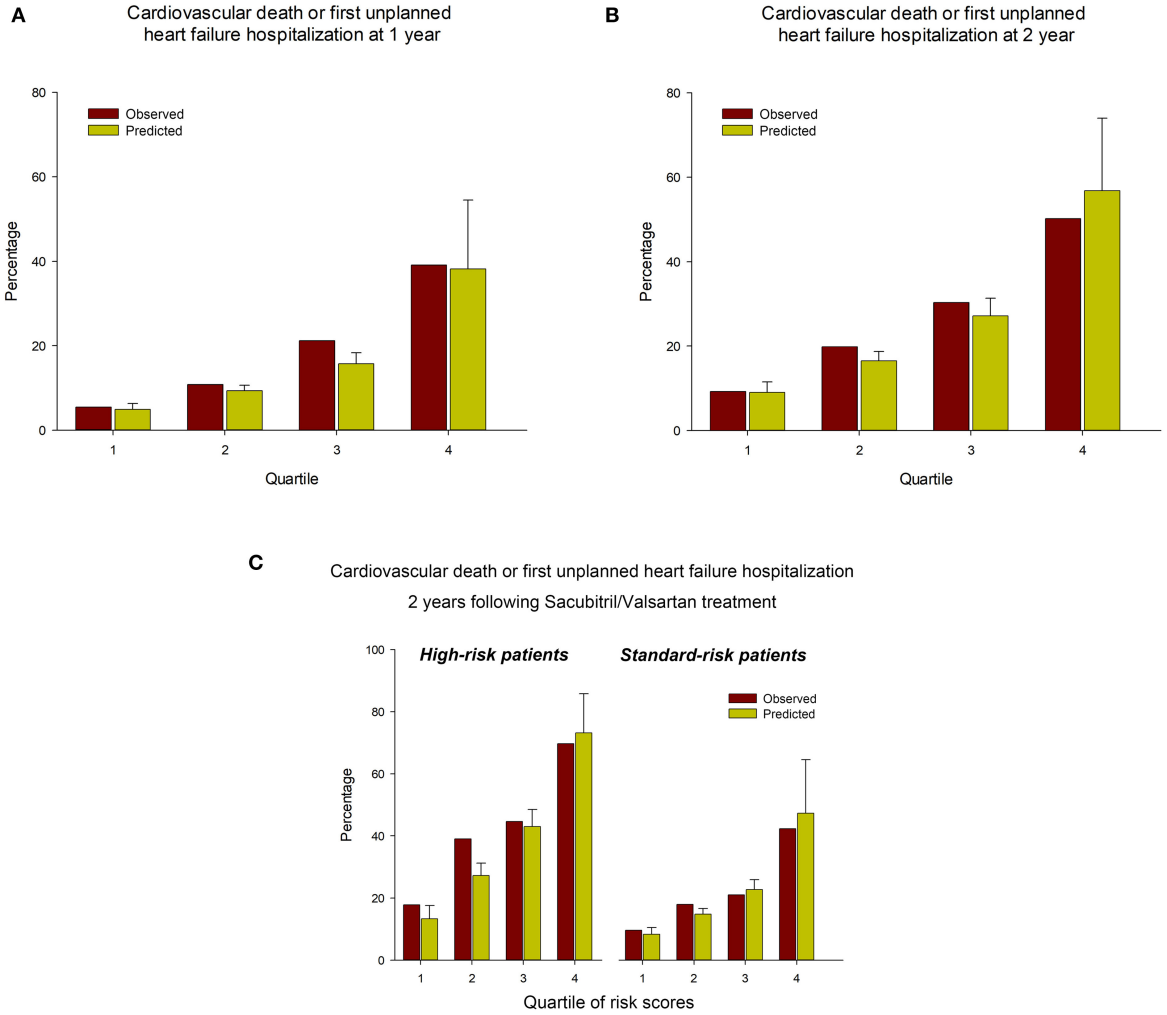
Comparisons between the predicted and observed probabilities of cardiovascular death or first unplanned HFH across patient risk quartiles at **(A)** 1 year and **(B)** 2 years. **(C)** Predicted vs. observed probabilities of cardiovascular death or first unplanned HFH, stratified by high/standard-risk and quartile of PREDICT-HF risk score.

An online calculator for the PREDICT-HF model was used to calculate the risks of cardiovascular death or first unplanned HFH, and cardiovascular death alone. Figure 1D shows Kaplan–Meier plots of the observed event rate in the TAROT-CHF cohort for clinical outcomes, categorized by quartile of risk score. Figures 2A,B demonstrate comparisons between the predicted and observed probabilities of cardiovascular death or first unplanned HFH across patient risk quartiles at 1 and 2 years. The C statistics for the PREDICT-HF model when applied to the study cohort for cardiovascular death or first unplanned HFH at 1 and 2 years were 0.74 (95% CI 0.70–0.77) and 0.73 (95% CI 0.70–0.76), respectively. The C statistics for cardiovascular death alone at 1 and 2 years were 0.77 (95% CI 0.72–0.82) and 0.76 (95% CI 0.71–0.80), respectively.

Among the high-risk and standard-risk patients, the PREDICT-HF model could still predict outcomes accurately. The C statistics for the PREDICT-HF model in the overall cohort and high-risk group for cardiovascular death or first unplanned HFH at 1 years were 0.74 (95% CI 0.70–0.77) and 0.73 (95% CI 0.67–0.79), and at 2 years were 0.73 (95% CI 0.70–0.76) and 0.71 (95% CI 0.65–0.76), respectively. Figure 2C demonstrates the predicted vs. observed probabilities of cardiovascular death or first unplanned HFH, stratified by high/standard-risk and quartile of PREDICT-HF risk score.

### Drug titration and discontinuation

The mean daily dose at the initiation of Sac/Val treatment was 116 ± 56 mg/day. Among the patients taking Sac/Val at the end of follow-up, the mean daily dose was 157 ± 87 mg/day.

**Figure 3:**
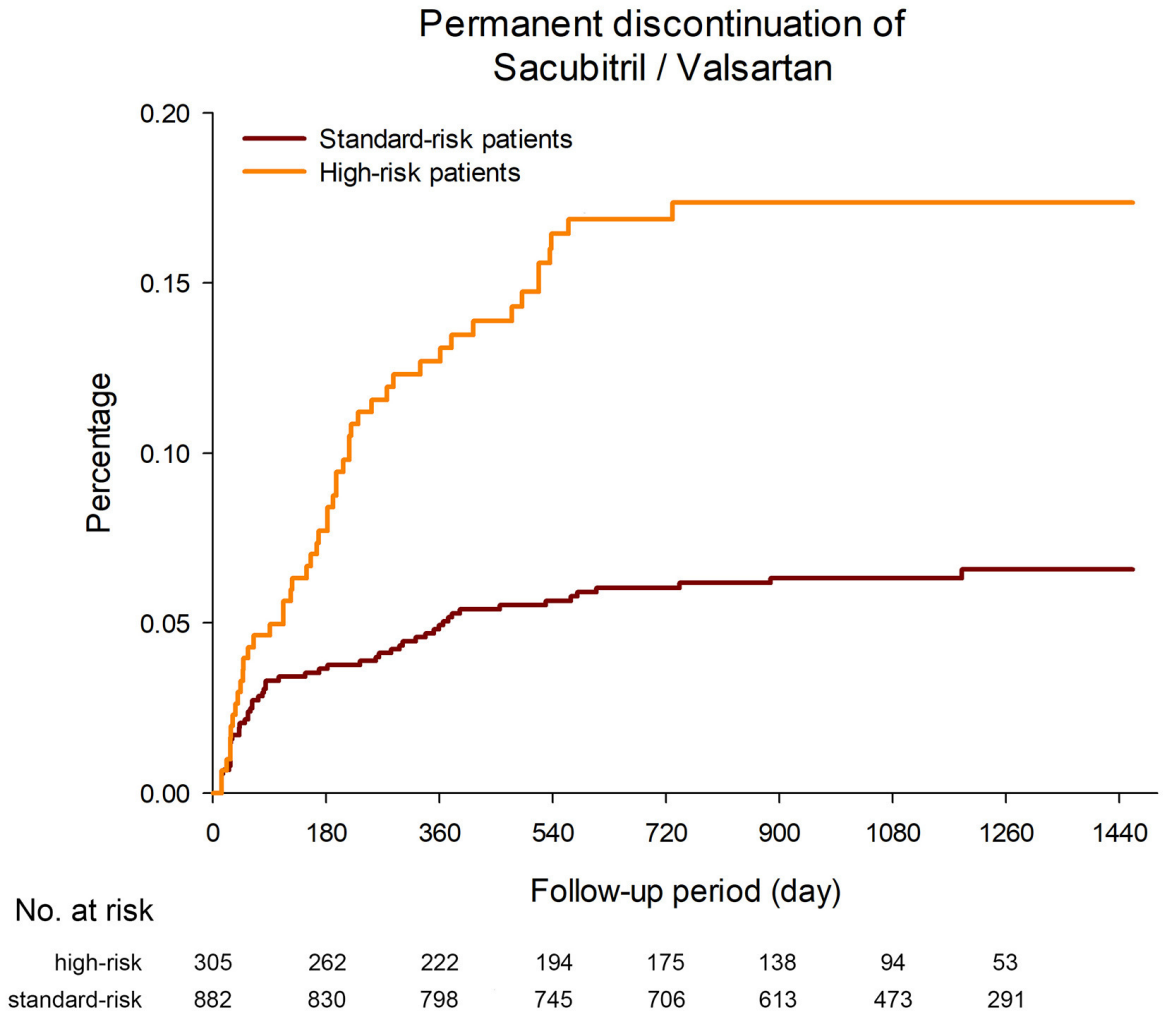
The permanent discontinuation rate among the high-risk patients was significantly higher than that among the standard-risk patients (8.3 vs. 2.5 discontinuation events per 100 patient-years, *p* < 0.001).

A total of 114 patients permanently discontinued Sac/Val during the follow-up period (3.7 discontinuation events per 100 patient-years). The median period from Sac/Val initiation to permanent discontinuation was 169 days (IQR 42–354 days). The permanent discontinuation rate among the high-risk patients was significantly higher than that among the standard-risk patients (8.3 vs. 2.5 discontinuation events per 100 patient-years, *p* < 0.001, Figure 3). Another 18 patients in the study discontinued Sac/Val transiently. Among these 132 patients who discontinued Sac/Val, the reasons for discontinuation were hypotension in 50 patients (37.9%), renal impairment or hyperkalemia in 26 patients (19.7%), adverse effects or allergy in 18 patients (13.6%), and others in 38 patients (28.8%).

## Discussion

In the present study, 1,187 patients were enrolled during the first 2 years after the introduction of Sac/Val into Taiwan. During a median follow-up period of 36.7 months, the main findings were that the effectiveness and side-effect profile were comparable to those observed in the PARADIGM-HF trial. Although the efficacy and safety of Sac/Val for the treatment of HFrEF patients have been shown in global clinical trials, patient profiles and outcomes vary between different countries (19). This highlights the need of country-based real-world data to better understand local patient and healthcare system's characteristics.

Clinical trials have strict inclusion and exclusion criteria, whereas real-world studies enroll non-selected patients. It is therefore difficult to compare the outcomes of randomized trials and the current study directly. In this single-arm, real-world, multicentric cohort, we classified our patients into high- and standard-risk subgroups. High-risk patients including octogenarians and those with hypotension or severe renal impairment are generally excluded from randomized trials. Our results showed that these high-risk patients had significantly higher risks compared with the PARADIGM-HF trial population. In contrast, among real-world patients younger than 80 years who met the blood pressure and renal function criteria of the PARADIGM-HF trial (standard-risk patients), the observed risks were comparable to those seen in the PARADIGM-HF trial (Table 2).

In this study, we found that 25.7% had a least one of the high risk profile. Considering the beneficial effect but higher cost of using Sac/Val over RASis for HFrEF patients, this real-world prescription pattern implies that in the beginning of Sac/Val introduction, physicians tend to switch traditional RASis to Sac/Val in patients with relatively poor condition. Moreover, we also found that Sac/Val was well-tolerated among our cohort of real-world HFrEF patients. During follow-up, only 9.6% of the TAROT-CHF patients discontinued treatment permanently. Among these patients, more than 50% discontinued Sac/Val within 6 months, indicating that acute hemodynamic change should be closely monitored after the initiation of Sac/Val. Similar to our findings, a previous real-world study reported that 5.5% of their patients discontinued Sac/Val during the first 6 weeks of treatment (20). Symptomatic hypotension developed in 14% of the patients receiving Sac/Val in the PARADIGM-HF trial, but only 0.9% of the patients permanently discontinued Sac/Val during the study period. This could be because the patients who were randomized were already “selected” after the run-in period, and were therefore more likely to tolerate Sac/Val. Several factors were associated with a higher risk of medication discontinuation in the PARADIGM-HF trial, including higher natriuretic peptide levels, lower blood pressure, eGFR < 60 ml/min/1.73 m^2^, and an ischemic cause (21). In the current study, it is reasonable to expect that the high-risk patients had a significantly higher risk of permanent Sac/Val discontinuation than standard-risk patients due to their old age, low systolic blood pressure or low eGFR. According to *post-hoc* analysis of the PARADIGM-HF study, patients who received sub-target doses of Sac/Val due to intolerance had similar benefit to those who tolerated higher doses (22), suggesting that even in patients who discontinue this drug, physicians should try to re-initiate Sac/Val at a lower dose in order to obtain clinical benefits. In the current study, 13.6% of the patients who discontinued Sac/Val were able to re-initiate the drug.

Predicting the risk in HFrEF patients may allow physicians to make accurate decisions regarding the timing of guideline-recommended medical therapy adjustments and referral for advanced HF treatment. Several models have been established for predicting adverse outcomes in patients with HF (23, 24). However, most of these models did not include natriuretic peptide in the derivation model and were developed before the era of Sac/Val. The PREDICT-HF model was developed according to the PARADIGM-HF cohort and is the most current predictive model (14). This model was externally validated for all-cause mortality by the SwedeHF registry (14, 25). Although the current study enrolled high-risk patients not included in the PARADIGM-HF trial, our results clearly demonstrated that the PREDICT-HF model performed well in real-world Asian HFrEF patients. In addition, our results showed that three easily detected clinical profiles of age, renal function, and systolic blood pressure could help to identify patients at risk before initiating Sac/Val. Nevertheless, the PREDICT-HF score model could distinguish future outcomes more accurately, as shown in Figure 2.

Several limitations inherent in the retrospective design of this study should be mentioned. First, treatment decisions were based on real-world practice by the participating cardiologists. This type of retrospective study may have potential unmeasured bias, however, the objective of this study was to include a broad range of patients reflecting the current reality of real-world practice for Sac/Val rather than the narrowly defined HFrEF population in clinical trials. Second, although the baseline characteristics of the current cohort were complete without missing data for the PREDICT-HF model, some laboratory data such as total bilirubin and natriuretic peptide were not available in 30–40% of the patients.

In conclusion, among a real-world Asian population with chronic HFrEF, the PREDICT-HF score model performed well in this Asian cohort receiving contemporary HF treatment. Three easily detected clinical profiles of age, renal function, and systolic BP could help to identify patients at risk before initiating Sac/Val.

## Data availability statement

The raw data supporting the conclusions of this article will be made available by the authors, without undue reservation.

## Ethics statement

The studies involving human participants were reviewed and approved by MacKay Memorial Hospital IRB (reference number: 18MMHIS115e), Taipei Medical University Hospital IRB (reference number: TMU-JIRB_N20204142), Cheng Hsin General Hospital IRB (reference number: (615)106A-23), and Chi-Mei Medical Center IRB (reference number: 10903-010).

## Author contributions

Conception, study design, data analysis, and interpretation: C-YH, H-YC, C-JC, and Y-HL. Project administration and data acquisition: C-YH, H-YC, W-RC, P-LL, F-PC, W-YL, J-LH, H-WL, C-TL, and Y-HL. Statistical analysis: H-YC and C-JC. Writing—original draft: C-YH and H-YC. Writing—review editing: C-JC and Y-HL. Funding acquisition: C-YH, H-YC, and Y-HL. All authors contributed to the article and approved the submitted version.

## Funding

The present work was supported by the following research grants: Taiwan Society of Cardiology (TSOC 107-0505), Cheng Hsin General Hospital (CHGH111-(N)09; CHGH111-(N)10), Taipei Medical University and Taipei Medical University Hospital (109TMU-TMUH-16, 110TMU-TMUH-14, 111TMUH-MOST-21), and the Ministry of Science and Technology (MOST-110-2314-B-038-131). The funding institutions took no part in the study design, data collection or analysis, publication intent, or manuscript preparation.

## Conflict of interest

The authors declare that the research was conducted in the absence of any commercial or financial relationships that could be construed as a potential conflict of interest.

## Publisher's note

All claims expressed in this article are solely those of the authors and do not necessarily represent those of their affiliated organizations, or those of the publisher, the editors and the reviewers. Any product that may be evaluated in this article, or claim that may be made by its manufacturer, is not guaranteed or endorsed by the publisher.
